# MMnc: multi-modal interpretable representation for non-coding RNA classification and class annotation

**DOI:** 10.1093/bioinformatics/btaf051

**Published:** 2025-01-31

**Authors:** Constance Creux, Farida Zehraoui, François Radvanyi, Fariza Tahi

**Affiliations:** Université Paris-Saclay, Univ Evry, IBISC, Evry-Courcouronnes 91020, France; Molecular Oncology, PSL Research University, CNRS, UMR 144, Institut Curie, Paris 75248, France; Université Paris-Saclay, Univ Evry, IBISC, Evry-Courcouronnes 91020, France; Molecular Oncology, PSL Research University, CNRS, UMR 144, Institut Curie, Paris 75248, France; Université Paris-Saclay, Univ Evry, IBISC, Evry-Courcouronnes 91020, France

## Abstract

**Motivation:**

As the biological roles and disease implications of non-coding RNAs continue to emerge, the need to thoroughly characterize previously unexplored non-coding RNAs becomes increasingly urgent. These molecules hold potential as biomarkers and therapeutic targets. However, the vast and complex nature of non-coding RNAs data presents a challenge. We introduce MMnc, an interpretable deep-learning approach designed to classify non-coding RNAs into functional groups. MMnc leverages multiple data sources—such as the sequence, secondary structure, and expression—using attention-based multi-modal data integration. This ensures the learning of meaningful representations while accounting for missing sources in some samples.

**Results:**

Our findings demonstrate that MMnc achieves high classification accuracy across diverse non-coding RNA classes. The method’s modular architecture allows for the consideration of multiple types of modalities, whereas other tools only consider one or two at most. MMnc is resilient to missing data, ensuring that all available information is effectively utilized. Importantly, the generated attention scores offer interpretable insights into the underlying patterns of the different non-coding RNA classes, potentially driving future non-coding RNA research and applications.

**Availability and implementation:**

Data and source code can be found at EvryRNA.ibisc.univ-evry.fr/EvryRNA/MMnc.

## Graphical abstract

**Graphical Abstract:**
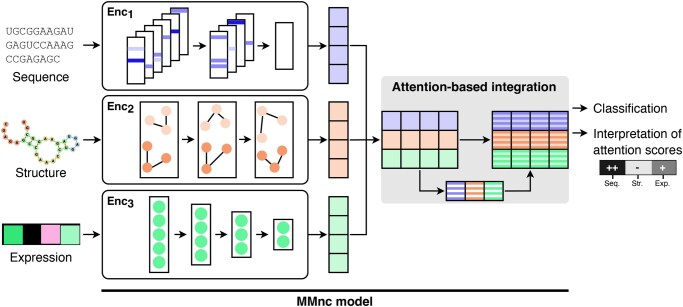

## 1 Introduction

Genome sequencing projects in the early 2000s transformed our understanding of eukaryotic genomes, revealing a vast number of non-coding genes. It is now estimated that 98.5% of the human genome is non-coding (Mattick 2001). Recently, numerous studies have demonstrated the critical roles non-coding RNAs (ncRNAs) play in normal cellular processes and disease development. Certain ncRNAs exhibit unique characteristics, such as stability in the bloodstream and high tissue specificity (Slack and Chinnaiyan 2019). As a result, the study of ncRNAs could lead to the discovery of novel biomarkers and therapeutic targets.

NcRNAs have two main types of functions: housekeeping and regulation. Different classes of ncRNAs sharing similar functions have been described for each type (Zhang *et al.* 2019). Housekeeping RNAs were discovered in the mid-1950s. They are essential for survival and are often involved in cell maintenance (Joshi *et al.* 2022). The main classes of housekeeping RNAs are ribosomal and transfer RNAs (rRNAs and tRNAs), both involved in translation, small nuclear RNAs (snRNAs), which intervene in messenger RNA (mRNA) splicing, and small nucleolar RNAs (snoRNAs), implicated in RNA modifications. Regulatory ncRNAs were only discovered in the 1990s and are still being investigated. They regulate gene expression through interactions with other molecules at epigenetic, transcriptional, and post-transcriptional levels. Their expression varies across tissues and conditions (de Goede *et al.* 2021). Classes include micro-RNAs (miRNAs), which are the most abundant and can bind multiple mRNAs, and small interfering RNAs (siRNAs), which also regulate expression by binding mRNAs but are specific to one target. Long non-coding RNAs (lncRNAs) are another class that can regulate expression by interacting with DNA, RNA, or proteins. They are mainly characterized by the length of their sequence, which can reach hundreds of kilobases.

In computational biology, classifying ncRNAs into functional groups allows for large-scale characterization of their functions, a process that would be both time-consuming and expensive if done experimentally. These functional groups can be defined at multiple levels, including families, which are precise groups of ncRNAs sharing a common evolutionary origin, and classes, which are broader, higher-level groups of ncRNAs with similar roles and that can encompass multiple families. Our work focuses on the latter level. Over the years, various methods have been developed to categorize ncRNAs into established functional classes. Early computational methods for ncRNA classification rely on probabilistic models, dynamic programming, or standard machine learning (ML) algorithms. As input, many methods use secondary structure motifs (Panwar *et al.* 2014) or sequence alignment (Nawrocki and Eddy 2013). With the development of new sequencing technologies, several methods proposed around 2010 use as input expression data from short RNA-sequencing experiments, and implement alignment-based methods or ML algorithms for classification (Fasold *et al.* 2011). The first application of deep learning (DL) came as a breakthrough in the field in 2017 and was adopted by later methods. The designed neural networks primarily consist of Convolutional Neural Networks (CNNs), Recurrent Neural Networks (RNNs), or hybrids that combine elements of both. The vast majority of methods use ncRNA sequences that are one-hot encoded as input to the DL model. This is the case of most methods, e.g. ncrna-deep (Noviello *et al.* 2020), MFPred (Chen *et al.* 2023), and NCYPred (Lima *et al.* 2023). BioDeepFuse (Avila Santos *et al.* 2024) combines this representation with hand-crafted features extracted from the sequence. The secondary structure is used as input by RNAGCN (Rossi *et al.* 2019), nRC (Fiannaca *et al.* 2017), and the method proposed by Sutanto and Turcotte (2023). In RNAGCN, structures are represented as graphs and processed by a Graph Neural Network (GNN). In the last two methods, a matrix representation is created, with features indicating the presence of different structural motifs. Only two methods use two types of data: imCnC (Boukelia *et al.* 2020) and ncRDense (Chantsalnyam *et al.* 2021). The first includes the sequence (one-hot encoded) as well as four different epigenetic marks (represented as matrices). In ncRDense, the sequence and the structure are used, both one-hot encoded separately. In both methods, inputs are first processed independently, and representations are then merged before classification. A detailed comparison of DL-based ncRNA classifiers can be found in Creux *et al.* (2024).

NcRNAs are complex molecules that can be described at different levels: their sequence, structure, expression, all carry complementary information. These points of view can be called modalities. State-of-the-art tools consider at most two types of modalities, and a large majority only exploits one, thus potentially ignoring important information. Moreover, we note that state-of-the-art tools are not interpretable. The study of ncRNAs is at the center of interactions between biology, medicine and computer science. In this context, providing interpretable results is important, as it facilitates information exchange.

We propose a multi-modal interpretable deep learning method called MMnc. The model integrates multiple modalities to describe ncRNAs, to take into account their various characteristics at different levels: sequence, secondary structure, and expression. The aims of the approach are to perform classification, and to provide interpretability in order to gain a deeper understanding of the characteristics of ncRNA classes. More precisely, multi-modal integration in MMnc is based on an attention mechanism to quantify the importance of the different modalities for each class. One key aspect of the proposed approach is its modularity. The number of modalities used for a dataset can be chosen depending on their availability. While state-of-the-art tools consider at most two modalities, our method is able to consider more, which provides a richer basis for classification and understanding of ncRNA classes. Moreover, MMnc handles missing data when a modality is present in a dataset but not for all samples, thus enabling the utilization of all available data.

This paper is organized as follows: Section 2 presents a description of our proposed method. In Section 3, we report the results of several experiments, including a comparison of different modality encoders, an ablation study, experiments with missing modalities, an illustration of the interpretability of the method, and the evaluation of MMnc’s performance against state-of-the-art tools. Finally, we outline potential directions for future works.

## 2 Materials and methods

In multi-modal deep learning approaches, one key choice is the method used to combine the different modalities. This step is called modality integration or fusion. There are three main integration strategies: early, late, and intermediate (Stahlschmidt *et al.* 2022). In early integration, modalities are concatenated before being input into the network. The drawback is that intra-modality interactions might not be sufficiently exploited. In late integration, separate models are trained for each modality, and the decisions returned by each model are fused. In this approach, complementarity between data is ignored. Intermediate integration has emerged as a good compromise, which leads to its frequent application (Choi and Lee 2019). Modalities are first processed by independent networks, returning latent representations that are fused before undergoing joint processing. Modalities can be fully exploited separately, and their complementarity is taken into account for the decision. Attention mechanisms, which have become exponentially more popular over the last decade (Bahdanau *et al.* 2016), have also been applied to multi-modal contexts with success (Yu *et al.* 2019). Cross-attention is used to compare data from multiple inputs and capture correlations between features across different modalities (Moon and Lee 2022). It allows the modeling of the relative importance of features and modalities, and provides interpretability of the model. Our approach is based on intermediate integration, and includes cross-attention for interpretability.

Our contribution is 2-fold. First, we introduce a novel interpretable architecture for multi-modal integration that is based on an attention mechanism. This approach is generic and can be used in multiple contexts, for different data types and downstream tasks. Second, we instantiate this approach for the classification of ncRNAs with three modalities: sequence, secondary structure, and expression. We choose appropriate modality representations, and design different encoding architectures for each modality. These contributions are detailed separately in the following sections.

### 2.1 Attention-based multi-modal integration

**Figure 1.**
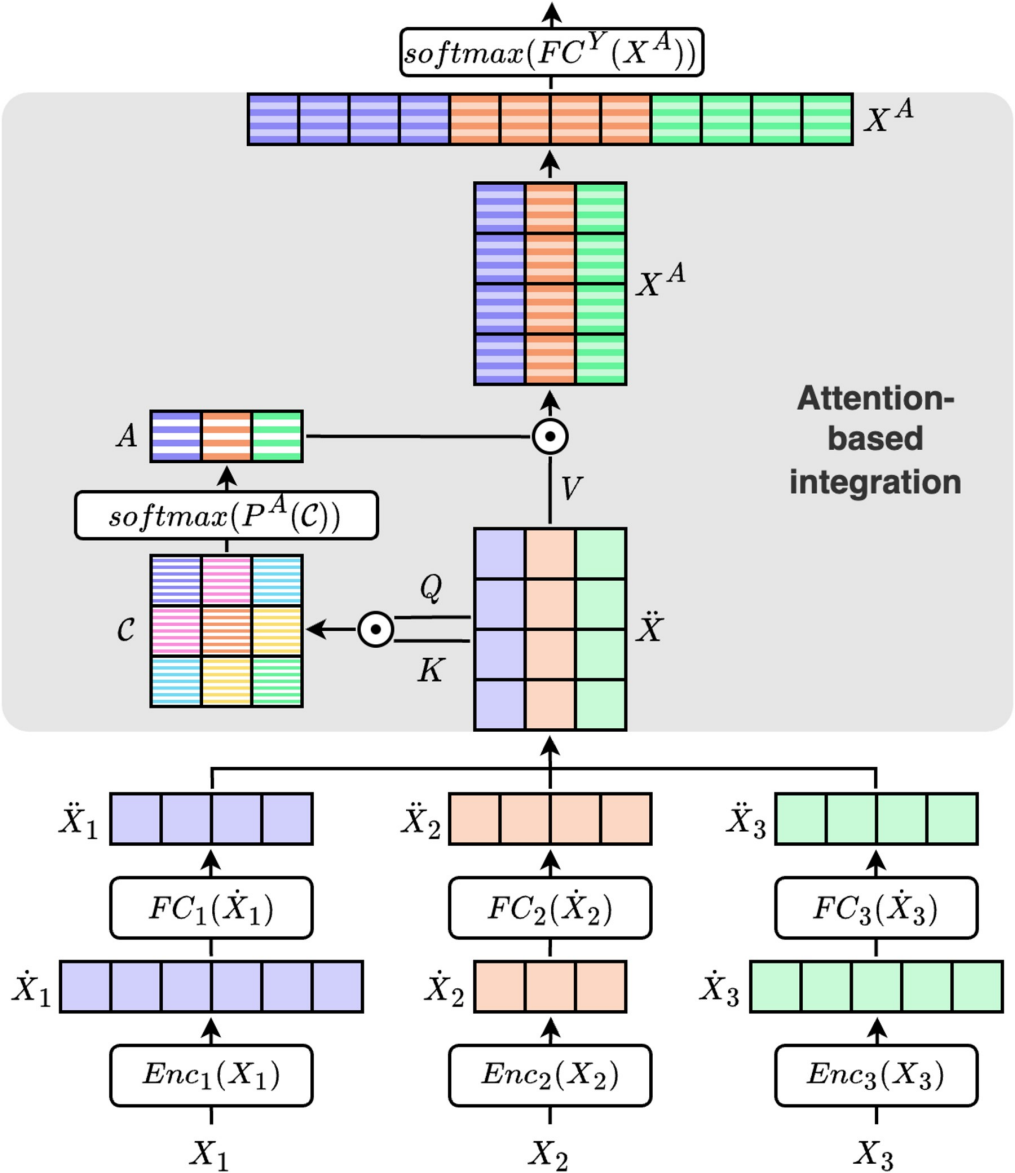
Architecture of the proposed method. $X_{1}$, $X_{2}$, and $X_{3}$ are example modalities. They are encoded separately. Then, cross-modal attention is computed and projected to obtain modality attention scores. These are used to weigh modalities in the integrated representation, which is used for classification.

The architecture of our method, illustrated in Fig. 1, can be separated into four main parts. The first is modality encoding, wherein each modality is transformed by an appropriate model to extract meaningful information. Then, the obtained modality representations are integrated using attention. At this step, scores of importance are associated with each modality. Our integration approach enables the model to handle missing modalities. After the integration step, we obtain a new representation of samples, which can be used for a downstream task, in our case classification. Training is performed end-to-end.

#### 2.1.1 Modality encoding

Let x∈X, be a sample represented by m modalities $x=[x_{1},\ldots ,x_{M}]$. Let $Enc_{m}$ be the model chosen to encode a modality m (1≤m≤M) in order to extract relevant information. We represent the encoded modality by ${\dot{x}}_{m}=Enc_{m}(x_{m})$, where ${\dot{x}}_{m}\in R^{D_{m}}$ ($D_{m}$ is the dimension of the encoded modality m).

#### 2.1.2 Attention-based integration

Modality representations are transformed to belong to a same-dimension space: they are projected into a feature space of dimension d with a fully connected layer as follows:

(1) $${\overset{¨}{x}}_{m}=FC_{m}({\dot{x}}_{m})=W_{m}\cdot {\dot{x}}_{m}+b_{m}$$

where ${\overset{¨}{x}}_{m}\in R^{D}$ is the obtained representation of a sample x for the modality m. $W_{m}\in R^{D\times D_{m}}$ and $b_{m}\in R^{D}$ are, respectively, the weights and biases of the projection.

Modality representations are then concatenated into a matrix $\overset{¨}{x}=[{\overset{¨}{x}}_{1},\ldots ,{\overset{¨}{x}}_{M}]$, where $\overset{¨}{x}\in R^{D\times M}$. $\overset{¨}{x}$ is converted into three matrices Q, K, $V\in R^{D\times M}$ representing, respectively, the query, key, and value for attention. Each is obtained using a specific fully connected layer: $Q=FC_{Q}(\overset{¨}{x})$, $K=FC_{K}(\overset{¨}{x})$, and $V=FC_{V}(\overset{¨}{x})$. We compute the dot product between Q and K normalized by the squared root of the dimension *d* to obtain a cross-modal and self-modal interaction matrix $C\in R^{M\times M}$, $C={\left(C_{nm}\right)}_{\left(1\leq n,m\leq M\right)}$:

(2) $$C_{nm}=\frac{Q_{m}^{T}\cdot K_{n}}{\sqrt{D}}$$

$C_{nm}$ corresponds to the cross-attention that represents how much the query modality m should focus on the key modality n ($Q_{m},K_{n}\in R^{D}$ are respectively the *m*th and *n*th columns of C). When n=m, $C_{mm}$ corresponds to a self-attention computed on the modality m. It captures the intra-modality relationship.

This interaction matrix is then transformed by a Perceptron $P^{A}(C)=W^{A}\cdot C+b^{A}$ to learn the combination scores of the m modalities. $W^{A}\in R^{1\times M}$ and $b^{A}\in R$ are, respectively, the weights vector and the bias of the Perceptron.

The resulting values represent each modality’s importance, including its interaction with other modalities.

A softmax function is used to return the final attention coefficients: $A=\mathrm{softmax}(P^{A}(C))$, where $A={\left(A_{m}\right)}_{\left(1\leq m\leq M\right)}$ is the attention vector associated with the m modalities.

The value V is weighted using attention coefficients, which are then flattened into a vector to obtain the final representation $x^{A}$, a row vector of dimension (M·D):

(3) $$x^{A}=[A_{1}\cdot V_{1},\ldots ,A_{M}\cdot V_{M}]$$

#### 2.1.3 Handling missing modalities

The proposed attention mechanism allows us to easily handle missing modalities. A mask is applied to set attention coefficients to zero for missing modalities. This is a simple way to ignore missing information, without introducing bias that traditional imputation methods like mean or median imputation can cause, and without the additional cost of more complex approaches like modality dropout (Cheerla and Gevaert 2019).

#### 2.1.4 Classification

The learned representation of modalities can be used for different downstream tasks. We are interested in classification problems, so we use a series of H fully connected layers (1≤h≤H), with the final layer returning class predictions, as follows:

(4) $$x^{h}=\mathrm{ReLU}(FC^{h}(x^{h-1}))=\mathrm{ReLU}(W^{h}\cdot x^{h-1}+b^{h})$$

 

(5) $$\hat{Y}=\mathrm{softmax}(FC^{Y}(x^{H-1}))=\mathrm{softmax}(W^{Y}\cdot x^{H-1}+b^{Y})$$

where $x^{0}=x^{A}$, W and b are the weights and biases of the layer.

The function used to train the model is the cross-entropy loss, which computes the difference in distribution between ground-truth labels Y and predicted labels $\hat{Y}$: $L=-\sum_{k=1}^{K}Y_{k}\;\text{log}(\hat{Y_{k}})$.

### 2.2 Model instantiation for non-coding RNA classification

**Figure 2.**
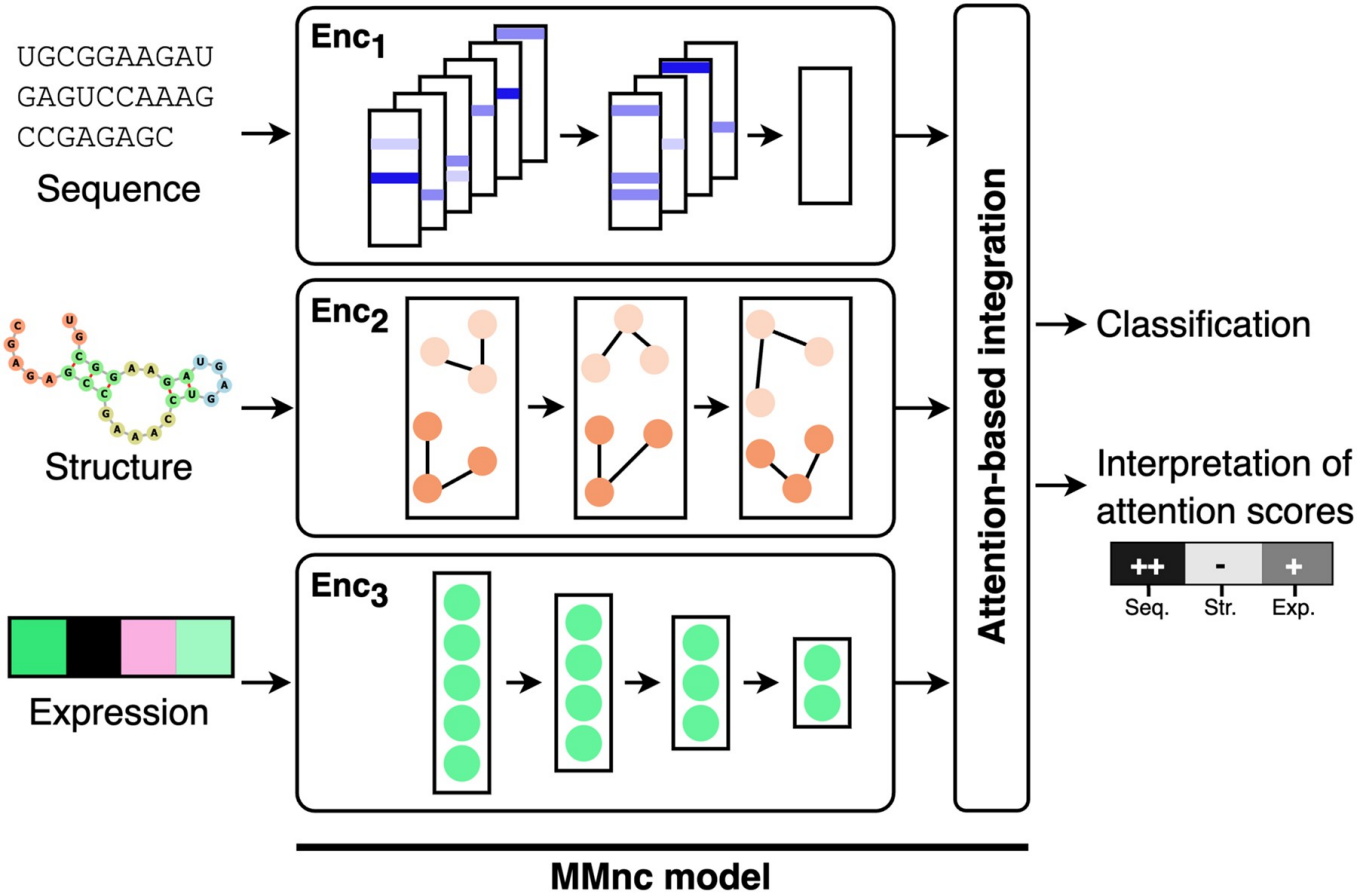
Illustration of our method MMnc for ncRNA classification. Three modalities are used: sequence, structure, and expression. First, each modality is processed separately to extract relevant representations. Then, these are integrated with a process based on attention. The sample is thus represented by information from all modalities. Classification is performed. Moreover, attention scores are returned and can be interpreted.

We propose MMnc, an interpretable multi-modal classifier that is based on the approach presented in the previous section, instantiated for non-coding RNA classification. Its architecture is illustrated in Fig. 2.

Three modalities are leveraged to represent ncRNAs: their sequences, secondary structures, and expression. The used modalities are characterized by the type of data they provide, so it is important to use appropriate encoders for each modality. We include three types of data: text (sequences), graph (secondary structures), and tabular (expressions).

#### 2.2.1 Sequence

NcRNA sequences are chains composed mainly of the four nucleotides A, C, G and U, but degenerate nucleotides can also be present. These sequences can be divided into k-mers, which are groups of k nucleotides.

Multiple types of models can be used to learn from textual data. In ncRNA classification literature, both RNNs and CNNs have been used, with CNNs performing better as shown by Noviello *et al.* (2020) and in our previous work (Creux *et al.* 2024). Transformers (Vaswani *et al.* 2017) have revolutionized natural language processing, but have not yet been used for ncRNA classification. We compare the use of CNNs to Transformers. We implement CNNs based on the following typical architecture: multiple convolutional blocks, each composed of a convolutional layer (1D) with leaky ReLU activation, batch normalization, max pooling, and dropout. These are followed by a flattening operation and blocks that consist of a fully connected layer and the same activation, normalization, and dropout. Training transformers requires extremely large amounts of data, which we consider too computationally expensive in the scope of our experiments. We instead perform transfer learning, using as a source the model DNABERT (Ji *et al.* 2021), which is based on BERT (Devlin *et al.* 2019) and is trained on DNA. While other Transformers adapted for nucleotidic sequences exist [Nucleic Transformer (He *et al.* 2023), RNABERT (Akiyama and Sakakibara 2022), DNABERT-2 (Zhou *et al.* 2023)], DNABERT performed best in preliminary comparisons and for other RNA-related tasks in our team (Bernard *et al.* 2025).

#### 2.2.2 Secondary structure

The secondary structure is the 2D arrangement of an RNA molecule, indicating how nucleotides pair with each other to form base pairs. These base pairs create specific patterns such as hairpins, loops, bulges, and stems, which are essential for the RNA’s function. The secondary structure is a step above the simple sequence of nucleotides and provides insight into how the RNA will fold in 3D and interact with other molecules. Because experimental data is scarce, secondary structures can be predicted using tools. We predict the secondary structure using MXfold2 (Sato *et al.* 2021), as it is one of the tools presenting the best compromise between accuracy and speed (Bugnon *et al.* 2022). While using a structure prediction algorithm able to handle non-canonical base pairs and pseudoknots would be interesting, current tools require extremely high prediction times. The secondary structure can be represented as a graph, in which nodes correspond to nucleotides. The edges are the bonds between adjacent or paired nucleotides.

Representing structures as graphs allows us to input the entire secondary structure into the network and extract relevant features. Adopting other approaches would lead to information loss. For example, treating the dot-bracket representation of the structure as a sentence that can be one-hot encoded to form a matrix, as can be done by other methods, leads to information loss as it cannot represent which two bases are paired together. In the literature, it is common to create a matrix of secondary structure motifs to avoid this problem. Still, this approach can be biased, as some important structural motifs might be missed. To address the mentioned issues, we explore the use of several variants of GNNs. In all cases, graph nodes are embedded based on nucleotide type, and edges are embedded based on whether they represent a bond between adjacent nucleotides or a base pairing. Five graph convolutional blocks are repeated, each including a graph convolution with leaky ReLU activation, batch normalization, and dropout. Then, Set2Set pooling (Vinyals *et al.* 2016) is applied.

#### 2.2.3 Expression

Expression data is obtained from RNA-sequencing experiments and reflects the level of expression of an RNA in different conditions. Therefore, a vector of normalized expression in different samples can be used to describe an RNA.

Earlier ncRNA classifiers used expression data as input, but deep learning models, which gained popularity more recently, have not been used. Instead, classifiers were typically based on Support Vector Machines, and were restricted to smaller datasets. While deep learning has not been used on expression data for ncRNA classification, it has proven successful for other expression-related tasks (Bourgeais *et al.* 2022, Beaude *et al.* 2023). In particular, the Multi-Layer Perceptron (MLP) is an architecture that is both simple and efficient to learn from expression data.

We implement MLPs that are composed of fully connected layers with ReLU activation, batch normalization, and dropout. Different numbers and sizes of hidden layers are tested.

## 3 Results and discussion

This section presents different experiments evaluating MMnc’s performance. First, we briefly present the three datasets used in the experiments. In Section 3.2, we compare the different strategies for modality encoding. Then, in Section 3.3, we perform an ablation study to show the importance of different choices in the model. In Section 3.4, we show how the results can be interpreted for class annotation. The performance of our method is compared to other state-of-the-art ncRNA classifiers in Section 3.5.

### 3.1 Datasets

We evaluate performance on three datasets: two from the state-of-the-art (D1 and D2), which only include the sequence and secondary structure, and a third dataset (D3) which is new. Further information on the composition of the datasets is given in Supplementary Table S1.

D1 was originally presented by Fiannaca *et al.* (2017). Some sequences are present in both the training and test sets, we remove them from the test set for our experiments. Moreover, we remove sequences with degenerate nucleotides, so the dataset can be used for all tools. It contains 8350 samples and 13 balanced classes: 5S rRNA, 5.8S rRNA, CD-box, HACA-box, Intron-gpI, Intron-gpII, IRES, leader, miRNA, riboswitch, ribozyme, scaRNA, tRNA.

D2 is obtained from Lima *et al.* (2023), contains 45 447 ncRNAs belonging to 13 classes. 11 are the same as in D1, but IRES and scaRNA are replaced by Y RNA and Y RNA-like.

D3 is a dataset we build using data from three popular RNA-seq cohorts: GTEx, TCGA and TARGET (Vivian *et al.* 2017). It contains transcript expression for 19 131 samples from cancerous and healthy tissues. The RNA classes and sequences of trancripts present in the expression data are obtained from Ensembl (Martin *et al.* 2023). We remove all protein-coding genes and pseudogenes, as well as some ncRNA classes due to low sample size. Four ncRNA classes remain: lncRNA, miRNA, snoRNA, and snRNA. We also filter out sequences longer than 1200 nt, which is similar to the maximum length in D1, and a manageable length for structure prediction tools. In the next step, we remove sequences exceed a 80% similarity threshold with the CD-HIT tool (Li and Godzik 2006). In total, this dataset contains 5929 ncRNAs ranging from 41 to 1200 nt. While the sequence and structure are present for all ncRNAs, 23% of ncRNAs lack expression measures for any tissue, in which case we consider the expression modality to be missing.

### 3.2 Comparison of different modality encoders

Choosing the appropriate encoder model for each modality is crucial to extract maximum information from the input data. We compare different encoders for each modality in Table 1.

**Table 1.** Comparison of accuracy for MMnc with different modality encoders.^a^

Modality	Model	D1	D2	D3
**Sequence**	DNABERT-3	0.922	0.975	0.975
	DNABERT-4	0.909	0.936	0.969
	DNABERT-5	0.925	0.963	0.966
	DNABERT-6	0.939	0.967	0.971
	CNN1	0.945	0.979	0.956
	CNN2	0.951	0.980	0.966
**Structure**	GIN, size 64	0.684	0.739	0.944
	GIN, size 128	0.748	0.802	0.932
	SAGE, size 64	0.759	0.831	0.944
	SAGE, size 128	0.797	0.807	0.925
	EdgeCNN, size 64	0.728	0.733	0.938
	EdgeCNN, size 128	0.776	0.83	0.903
**Expression**	MLP1, drop. 0.2			0.773
	MLP1, drop. 0.4			0.790
	MLP2, drop. 0.2			0.779
	MLP2, drop. 0.4			0.775

a Best values are underlined.

**Sequence.** We present two CNN variants with 5 convolutional blocks. For CNN1, their sizes are [128, 128, 256, 256, 256], and they are [256, 256, 256, 256, 256] in CNN2. The other parameters are the same: the kernel size is 10, dropout is set to 0.2, and the convolutions are followed by two fully connected blocks of sizes 128 and 64. For the Transformer model, we test existing DNABERT variants that learn from sequences transformed into 3-, 4-, 5-, or 6-mers. This model has a limit of 512 nt for sequence length. In the case of longer sequences, only the first 512 nt are considered.

While accuracies for the different sequence encoders are close, the Transformers require more computational time and resources. The best model overall seems to be the proposed CNN2.

**Secondary structure.** The different GNNs implemented are based on three types of graph convolutions: GIN (Xu *et al.* 2019), SAGE (Hamilton *et al.* 2017), and EdgeCNN (Wang *et al.* 2019). We vary the size of the convolutions between 64 and 128, and set the dropout rate to 0.2. From the obtained results on the three datasets, SAGE convolutions seem to be the optimal choice for extracting information from RNA secondary structures, as they lead to the best results.

**Expression.** For expression encoders, we varied the number and size of blocks between the following MLP configurations: MLP1 has 3 blocks of sizes [2048, 512, 64], and MLP2 has 6 blocks of sizes [2048, 1024, 512, 256, 128, 64]. For each MLP variant, we compare 0.2 and 0.4 dropout. We observe that the best expression encoder on D3 is the configuration MLP1 with 0.4 dropout.

### 3.3 Ablation study

In the following, we demonstrate the use of different components of our method through an ablation study. We first compare the contribution of the different modalities (sequence, secondary structure, and expression) in the classification of ncRNAs. Then, we discuss the benefits of including attention in our model with regard to missing data.

#### 3.3.1 Contribution of modalities

We describe in Table 2 the results of ablation of the available modalities in our method for the three datasets. The first three rows show that all modalities can independently be used to classify ncRNAs, though with varying degrees of accuracy. The sequence is the most informative, followed by the structure, and finally, the expression. In the three next rows, we experiment with bi-modal combinations. The scores are either similar to or higher than uni-modal performances, and show that modalities provide complementary information. This is further illustrated by the performance of the model trained on the three modalities available in D3, which obtains the best scores for this dataset. Even when scores are similar, the use of multiple modalities is still relevant, as it can provide a new description of classes, and makes the inclusion of samples with missing modalities possible.

**Table 2.** Accuracy of MMnc for different modalities.^a^

Sequence	Structure	Expression	D1	D2	D3
X			0.951	0.980	0.966
	X		0.797	0.831	0.944
		X			0.790
X	X		0.953	0.981	0.977
X		X			0.973
	X	X			0.945
X	X	X			0.982

a We compare performance with different combinations of all available modalities.

**Figure 3.**
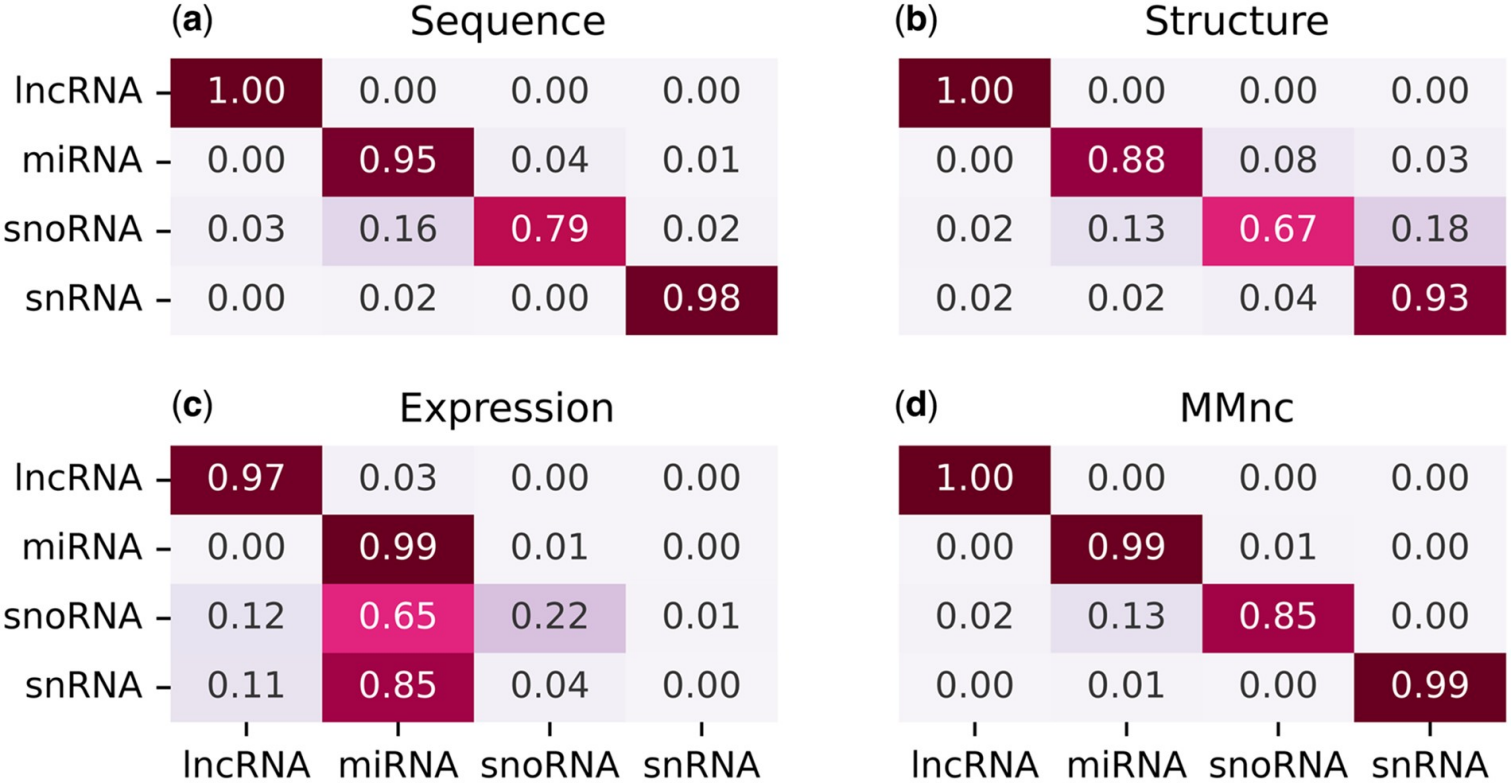
Confusion matrices on D3. Matrices (a–c) correspond to results of our model trained on one modality only, and matrix (d) presents results for the model trained on all modalities.

A better understanding of the difference in predictions between modalities can be gained by looking at the confusion matrices presented in Fig. 3. We notice that the expression-only model (c), which is the least accurate, is the one that captures the most useful information to classify miRNAs. Other classes are also better classified when all modalities are included. For example in the case of snoRNAs, the highest accuracy obtained by an uni-modal model is 0.79, but the multi-modal model reaches 0.85.

#### 3.3.2 Attention for missing modalities

The attention mechanism is integral to our model for two reasons. The first is interpretability. Unlike standard interpretation approaches that provide the most important features, our aim is to provide the most important sources without requiring post-hoc analyses. This property is suitable for ncRNA classification, as different modalities can be more or less characteristic for each class. The second is to handle missing data modalities. Data can be difficult to obtain for some ncRNAs: e.g. predicting the secondary structure of very long RNAs is not always possible; some ncRNAs might be filtered out of expression datasets due to their low expression level. The attention mechanism allows the model to learn to ignore missing modalities, and still make predictions based on the available data. Without attention in our model, we would either (i) have to remove a modality when data is missing for some samples, or (ii) need to remove samples if they have missing data. Both of these options would lead to a loss of information. Scenario (i) corresponds to the models trained without all modalities in Table 2. We note that removing modalities leads to worse performances: e.g. when MMnc is trained on all three modalities for D3, it reaches an accuracy of 0.982, which drops to 0.945 when the sequence is not used. In scenario (ii), we train MMnc on D3 with three modalities, but remove the 23% of samples that have missing data. The obtained accuracy is 0.889, a drop of 10% compared to the model trained on the dataset with missing modalities. Thus, learning from more samples is beneficial even when these have partially missing information. These two scenarios show that the model can still learn from incomplete data, and that the attention mechanism is a crucial component of our method.

### 3.4 Interpretation of attention for class annotation

**Figure 4.**
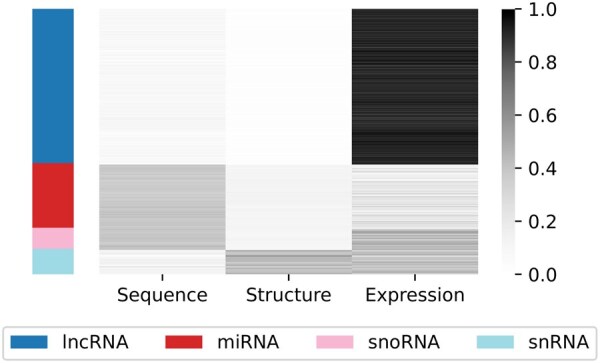
Visualization of modality attention scores in D3. Each row corresponds to a ncRNA from the dataset, with each column representing the importance given to each modality for the ncRNA’s classification. Darker values signify a stronger importance. NcRNAs are ordered by class, with the different classes represented on the left-hand side of the figure.

One of the key aspects of MMnc is its ability to describe the importance of different modalities in the classification of samples, through returning attention scores. By visualizing these attention scores, results can be exploited to further characterize the different ncRNA classes. We focus on D3 in this section, as it is the dataset with the most modalities. Figure 4 illustrates how MMnc can be used for class annotation, and shows that the different classes present in the dataset are characterized by different modalities.

We see that for lncRNAs, the defining modality seems to be expression. Compared to other ncRNA classes, lncRNAs are highly diverse, capable of binding different types of molecules, leading to varied sequence and structural patterns. However, multiple studies have highlighted their tissue-specific expression patterns, which could explain the model’s focus on expression. For miRNA prediction, the model primarily emphasizes the sequence. MiRNA precursors have a distinctive hairpin-loop structure, driven by specific sequence patterns, which the model likely captures. For snoRNAs, both sequence and expression are important. The CD-box and HACA-box snoRNA families are well-known for their strongly conserved sequence motifs. For snRNAs, structure and expression are underscored. The structural focus may reflect the presence of common protein-binding patterns, as snRNAs function within small nuclear ribonucleoprotein (snRNP) complexes.

### 3.5 Evaluation of performance against state-of-the-art tools

We measure the prediction performance of our method, MMnc, against six recent non-coding RNA classifiers. Four of them can be re-trained: RNAGCN (Rossi *et al.* 2019), MFPred (Chen *et al.* 2023), ncrna-deep (Noviello *et al.* 2020), and BioDeepFuse (Avila Santos *et al.* 2024). Two can only be used for prediction: ncRDense (Chantsalnyam *et al.* 2021) and NCYPred (Lima *et al.* 2023). These tools are chosen based on their high reported performance, and because sufficient guidelines are given for their execution. For RNAGCN, MFPred and ncrna-deep, hyperparameters are selected based on a previous study (Creux *et al.* 2024). For BioDeepFuse, the best hyperparameters presented in the publication are used. For our method, the sequence and secondary structure are used for all datasets. Expression is also used for D3.

D1 and D2 are presented with independent test sets (respectively, 26% and 30% of dataset size). For D3, we separate 20% of the dataset for testing, respecting class distribution. The rest of the datasets is used for training and validation. We perform 10-fold cross-validation, where each fold is stratified to ensure that class distribution is the same in the training and validation sets. Each sample appears in exactly one validation set.

We report in Table 3 the mean and standard deviation of performance metrics obtained over the 10 folds. We also present the performance on the test set. MMnc obtains the highest scores on all datasets. ncrna-deep’s results are also very high. However, unlike our method, ncrna-deep is not interpretable, and does not have the ability to handle missing data. What is more, while ncrna-deep is solely based on the sequence, our method integrates multiple modalities, which can be exploited in further work to better understand the characteristics of ncRNA classes. MMnc scores higher than the remaining methods on all datasets. RNAGCN, a tool solely based on the secondary structure, performs well on D2 and D3, but obtains lower scores on D1, which has less samples per class. BioDeepFuse and MFPred, both based on the sequence, perform better on D2 and D3, with MFPred having higher scores overall. ncRDense and NCYPred have fluctuating performances. This is because these tools can only be used in their pre-trained versions, so they are only able to predict classes that were present in their training sets. This explains why these methods’ scores are not consistent across datasets, and why performance is particularly poor on D3 which contains more classes unknown by the tools. ncRDense is the other multi-modal method in this benchmark, using the sequence and secondary structure. We base its comparison with our method on D1, for which ncRDense knows all classes and should obtain optimal performances. MMnc outperforms ncRDense, suggesting that the representation learned by our method captures more relevant information. One difference between both methods is that ncRDense uses a one-hot encoded representation of the secondary structure, while we represent it as a graph which, among others, has the benefit of better exploiting base pairing information.

**Table 3.** Comparison of performance of MMnc with state-of-the-art ncRNA classifiers.^a^

		(a)					(b)				
		Acc	MCC	F1	Pre	Rec	Acc	MCC	F1	Pre	Rec
**D1**	**RNAGCN**	0.851 ± 0.009	0.839 ± 0.010	0.848 ± 0.010	0.854 ± 0.010	0.847 ± 0.010	0.872	0.861	0.868	0.871	0.871
	**ncrna-deep**	0.914 ± 0.013	0.908 ± 0.014	0.913 ± 0.013	0.918 ± 0.012	0.912 ± 0.013	0.950	0.946	0.950	0.951	0.951
	**MFPred**	0.873 ± 0.020	0.863 ± 0.021	0.872 ± 0.020	0.879 ± 0.019	0.871 ± 0.020	0.907	0.899	0.911	0.916	0.909
	**BioDeepFuse**	0.804 ± 0.014	0.788 ± 0.016	0.802 ± 0.015	0.805 ± 0.015	0.804 ± 0.015	0.847	0.834	0.846	0.848	0.849
	ncRDense*	n/a	n/a	n/a	n/a	n/a	0.912	0.905	0.912	0.920	0.910
	NCYPred*	n/a	n/a	n/a	n/a	n/a	0.798	0.783	0.716	0.705	0.744
	**MMnc**	0.946 ± 0.008	0.942 ± 0.009	0.946 ± 0.008	0.949 ± 0.008	0.944 ± 0.009	0.953	0.946	0.952	0.955	0.949
**D2**	**RNAGCN**	0.945 ± 0.004	0.939 ± 0.004	0.940 ± 0.006	0.942 ± 0.008	0.940 ± 0.011	0.947	0.941	0.944	0.954	0.936
	**ncrna-deep**	0.970 ± 0.004	0.967 ± 0.004	0.974 ± 0.004	0.978 ± 0.002	0.971 ± 0.007	0.971	0.968	0.970	0.976	0.965
	**MFPred**	0.954 ± 0.011	0.949 ± 0.012	0.958 ± 0.010	0.964 ± 0.011	0.955 ± 0.009	0.965	0.961	0.968	0.974	0.962
	**BioDeepFuse**	0.910 ± 0.016	0.900 ± 0.018	0.889 ± 0.027	0.917 ± 0.029	0.875 ± 0.027	0.909	0.899	0.901	0.927	0.886
	ncRDense*	n/a	n/a	n/a	n/a	n/a	0.735	0.712	0.520	0.549	0.562
	NCYPred*	n/a	n/a	n/a	n/a	n/a	0.916	0.906	0.909	0.926	0.895
	**MMnc**	0.979 ± 0.001	0.977 ± 0.001	0.978 ± 0.004	0.982 ± 0.003	0.975 ± 0.006	0.981	0.978	0.983	0.985	0.980
**D3**	**RNAGCN**	0.952 ± 0.007	0.917 ± 0.011	0.898 ± 0.014	0.899 ± 0.017	0.898 ± 0.012	0.947	0.909	0.877	0.877	0.878
	**ncrna-deep**	0.964 ± 0.010	0.938 ± 0.017	0.922 ± 0.024	0.942 ± 0.020	0.910 ± 0.031	0.970	0.948	0.933	0.948	0.921
	**MFPred**	0.930 ± 0.014	0.882 ± 0.021	0.863 ± 0.020	0.872 ± 0.027	0.860 ± 0.023	0.957	0.926	0.912	0.908	0.916
	**BioDeepFuse**	0.943 ± 0.006	0.903 ± 0.011	0.875 ± 0.013	0.883 ± 0.012	0.870 ± 0.018	0.953	0.919	0.892	0.903	0.884
	ncRDense*	n/a	n/a	n/a	n/a	n/a	0.181	0.166	0.048	0.044	0.053
	NCYPred*	n/a	n/a	n/a	n/a	n/a	0.181	0.181	0.048	0.046	0.050
	**MMnc**	0.974 ± 0.006	0.964 ± 0.009	0.969 ± 0.008	0.973 ± 0.008	0.966 ± 0.008	0.982	0.970	0.964	0.973	0.956

a (a) Cross-validation scores (mean ± SD). (b) Test scores. Tools marked with an asterisk cannot not be re-trained, and their cross-validation results were not obtained (marked with “n/a”). Accuracy (“Acc”), MCC, F1-score (“F1”), precision (“Pre”), and recall (“Rec”) are reported (defined in Supplementary Equations S1–S5). The best results are underlined.

## 4 Conclusion

As interest in understanding the diverse functions of non-coding RNAs grows, we present MMnc, an interpretable multi-modal deep learning method for ncRNA classification. MMnc is a novel attention-based approach for intermediate modality integration. It creates a rich latent representation that captures both the unique information from each modality and the cooperation between them, in addition to effectively handling missing data. This leads to improved classification performance compared to the state-of-the-art, as demonstrated on three datasets. Crucially, MMnc also provides interpretability by assigning coefficients to each modality, shedding light on their relative contributions and enhancing our understanding of ncRNA classes.

Our method’s modular design ensures versality, allowing easy adaptation. For instance, the secondary structure prediction tool we currently use could be replaced by another if more accurate tools are proposed, perhaps including pseudoknots and non-canonical base pairings. Additionally, the framework can be extended to include other relevant modalities, such as tertiary structure or epigenetic marks, further enriching the characterization of ncRNA classes.

Future work will focus on expanding the scope of MMnc to explore inter-class similarities and the discovery of novel ncRNA classes. This extension could refine the existing classification frameworks, offering deeper insights into the diverse roles of ncRNAs in biological processes and disease mechanisms.

## Supplementary Material

btaf051_Supplementary_Data

## Data Availability

Data and source code can be found at EvryRNA.ibisc.univ-evry.fr/EvryRNA/MMnc.
