# Supplementary material for: MMnc: multi-modal interpretable representation for non-coding RNA classification and class annotation
Source: Bioinformatics. 2025 Jan 31;41(3):btaf051. doi: 10.1093/bioinformatics/btaf051 (PMC11890286; doi:10.1093/bioinformatics/btaf051)
Supplement: btaf051_Supplementary_Data [file btaf051_supplementary_data.pdf]

MMnc: Multi-modal interpretable representation for non-coding RNA classification and class annotation

Constance Creux[1,2], Farida Zehraoui[1], François Radvanyi[2], and Fariza Tahi[1]
[1] Université Paris-Saclay, Univ Evry, IBISC, 91020, Evry-Courcouronnes, France
[2] Molecular Oncology, PSL Research University, CNRS, UMR 144, Institut Curie, Paris, France

A. Datasets composition

Label	Train size	Test size
**D1**		
**5S rRNA**	489	192
**5.8S rRNA**	442	163
**CD-box**	496	191
**HACA-box**	498	193
**Intron gpI**	454	163
**Intron gpII**	488	155
**IRES**	312	113
**leader**	497	144
**miRNA**	496	194
**riboswitch**	498	193
**ribozyme**	496	186
**scaRNA**	498	102
**tRNA**	497	200
**Total**	6,161	2,189
**D2**		
**5S rRNA**	3,496	1,500
**5.8S rRNA**	322	126
**CD-box**	3,492	1,504
**HACA-box**	3,514	1,485
**Intron gpI**	903	390
**Intron gpII**	2,475	1,089
**leader**	3,481	1,514
**miRNA**	3,529	1,466
**riboswitch**	3,512	1,483
**ribozyme**	3,218	1,408
**tRNA**	3,463	1,533
**Y RNA**	320	107
**Y RNA-like**	76	41
**Total**	31,801	13,646
**D3**		
**lncRNA**	2,781	696
**miRNA**	1,154	288
**snoRNA**	375	94
**snRNA**	433	108
**Total**	4,743	1186

Table 1. Composition of D1, D2 and D3. The number of samples of the training and test sets is displayed for each class.

B. Metrics

$$\text{Accuracy} = \frac{1}{N}\sum_{k=1}^{K} TP_k \tag{1}$$

$$\text{MCC} = \frac{(\sum_{k=1}^{K} TP_k) * N - \sum_{k=1}^{K}(p_k * t_k)}{\sqrt{(N^2 - \sum_{k=1}^{K} p_k^2)(N^2 - \sum_{k=1}^{K} t_k^2)}} \tag{2}$$

$$\text{F1-score} = \frac{1}{K}\sum_{k=1}^{K}\frac{TP_k}{TP_k + 0.5(FP_k + FN_k)} \tag{3}$$

$$\text{Precision} = \frac{1}{K}\sum_{k=1}^{K}\frac{TP_k}{TP_k + FP_k} \tag{4}$$

$$\text{Recall} = \frac{1}{K}\sum_{k=1}^{K}\frac{TP_k}{TP_k + FN_k} \tag{5}$$

with K the number of classes and N the number of samples, TP_k, FP_k, TN_k, FN_k the True Positives, False Positives, True Negatives, False Negatives of class k; p_k is the number of times class k was predicted and t_k the number of members of class k.
